# Seminal plasma concentrations of long-acting cabotegravir in men receiving HIV pre-exposure prophylaxis

**DOI:** 10.1128/aac.00165-26

**Published:** 2026-04-15

**Authors:** Davide Moschese, Chiara Fusetti, Samuel Lazzarin, Andrea Giacomelli, Valentina Mazzotta, Dario Cattaneo, Antonio D’Avolio, Francesco Caruso, Donatella Maddalena, Francesco Petri, Andrea Antinori, Cristina Gervasoni, Andrea Gori, Maria Vittoria Cossu

**Affiliations:** 1 Department of Infectious Diseases, Luigi Sacco University Hospital, ASST Fatebenefratelli Sacco 18602 , Milan, Italy; 2 Department of Biomedical and Clinical Sciences, Università degli Studi di Milano 9304, Milan, Italy; 3 Regional AIDS Reference Center, National Institute for Infectious Diseases, Lazzaro Spallanzani IRCCS 18653 , Rome, Italy; 4 Department of Biomedical Sciences, Humanitas University 437807, Pieve Emanuele, Italy; 5 IRCCS Humanitas Research Hospital 9268, Milan, Italy; 6 Laboratory of Clinical Pharmacology and Pharmacogenetics, Department of Medical Sciences, University of Turin, Amedeo di Savoia Hospital 9314, Turin, Italy; 7 Health Direction, National Institute for Infectious Diseases, Lazzaro Spallanzani IRCCS 18653 , Rome, Italy; 8 Centre for Multidisciplinary Research in Health Science (MACH), Milan, Italy; Providence Portland Medical Center, Portland, Oregon, USA

**Keywords:** cabotegravir, long-acting PrEP, seminal plasma, pharmacokinetics, HIV prevention, genital-tract drug distribution

## Abstract

To address the lack of data on male genital tract penetration of CAB-LA PrEP, seminal plasma was collected at days 3, 7, 28, 84, and 140 and analyzed by LC–MS/MS. CAB was detectable in 71% of samples at day 3, 73% at day 7, and 92% at day 28, with all samples quantifiable at days 84 and 140. Seminal-to-blood plasma ratios remained low but proportional to systemic exposure, indicating sustained seminal CAB exposure.

## INTRODUCTION

Long-acting cabotegravir (CAB-LA) is an effective agent for HIV pre-exposure prophylaxis (PrEP); however, data describing its distribution into male genital secretions remain limited. Multicompartment pharmacokinetic studies conducted in healthy adults and in people with HIV (PWH) have shown that CAB reaches genital tissues and secretions at lower levels than in plasma. Nevertheless, these studies also consistently show that the pharmacologically active, unbound fraction remains sufficient to support meaningful antiviral activity at mucosal sites, consistent with established principles of genital-tract drug disposition (1–3). Additional evidence indicates that systemic CAB concentrations rapidly reach protective thresholds after initiation in people receiving long-acting injectable therapy (4, 5).

Despite these advances, CAB-LA concentrations in seminal plasma among PrEP users have not been previously evaluated. We therefore report the first characterization of CAB levels in seminal plasma among cisgender gay and bisexual men receiving CAB-LA for PrEP in a real-world scenario. We conducted a prospective pharmacokinetic study of individuals initiating CAB-LA for PrEP at the HIV/STIs Clinic of Luigi Sacco University Hospital (Milan, Italy) starting in January 2025. Eligible participants were individuals without HIV aged ≥18 years who initiated CAB-LA according to approved dosing: a 600 mg intramuscular injection administered into the gluteal muscle administered at baseline and again after 28 days, followed by injections every 56 days.

Seminal plasma samples for therapeutic drug monitoring (TDM) were collected at days 3, 7, 28, 84, and 140 after the first injection; days 28, 84, and 140 corresponded to pre-injection troughs. As this was an exploratory pharmacokinetic study conducted in a real-world clinical setting, no formal sample size calculation was performed. Sociodemographic and clinical data were obtained at baseline, and weight and height were measured at each visit. Participants were instructed to abstain from ejaculation for at least 48 h before sample collection. Semen samples were collected into sterile polypropylene containers, centrifuged to separate seminal plasma, and stored at −80°C until analysis.

CAB concentrations in seminal plasma were quantified by liquid chromatography–tandem mass spectrometry (LC–MS/MS). The assay lower limit of quantification (LLOQ) was 4 ng/mL; measurements ≤LLOQ were reported descriptively but excluded from summary analyses. Quality control samples met standard bioanalytical acceptance criteria (5). Seminal plasma concentrations were summarized as medians with interquartile ranges (IQRs) at each visit. Where applicable, results are expressed as regression coefficients (*β*) with 95% confidence intervals (CIs), representing log differences in geometric mean seminal concentrations. Exploratory analyses evaluated the association between body mass index (BMI) and seminal CAB exposure. Logistic regression models assessed the probability of detectable seminal concentrations at days 3, 7, and 28, while linear regression models evaluated the association between BMI and log-transformed seminal concentrations at days 84 and 140. Statistical significance was set at *P* < 0.05.

We enrolled 43 cisgender gay and bisexual men receiving CAB-LA for PrEP. The median age was 38 years (IQR: 33–42). Most participants identified as White/Caucasian, while five participants identified as non-White (Latin/Hispanic or Asian). At baseline, 24 participants (55.8%) had a body mass index (BMI) within the normal range (18.5–24.9 kg/m²), 15 (34.9%) were overweight (25.0–29.9 kg/m²), 3 (7%) were obese (≥30 kg/m²), and 1 (2.3%) was underweight (<18.5 kg/m²). At day 3 after the first injection, 21 seminal plasma samples were collected; 6 (28.6%) were below the LLOQ (Table 1). Among quantifiable samples (*n* = 15), median CAB concentration was 12 ng/mL (IQR: 6.5–23.5) with a seminal-to-blood plasma (SP:BP) ratio of 0.01 (IQR: 0.01–0.02). At day 7, 33 samples were collected; 9 (27.3%) were below the LLOQ. The median concentration was 16.0 ng/mL (IQR: 10–36) with a SP:BP ratio of 0.01 (IQR: 0.01–0.02). At day 28 (pre-second dose), 39 samples were collected; 3 (7.7%) below the LLOQ. Median concentration was 15.5 ng/mL (IQR: 10–41.25) with a SP:BP ratio of 0.01 (IQR: 0.01–0.02). At day 84 (pre-third dose), all 42 samples were quantifiable with a median concentration of 35 ng/mL (IQR: 23.25–49.75) and a SP:BP ratio of 0.02 (IQR: 0.02–0.03). At day 140 (pre-fourth dose), all 21 samples were quantifiable: median 31 ng/mL (IQR: 18–48) and ratio 0.02 (IQR: 0.015–0.03).

**TABLE 1:** Median cabotegravir concentrations (ng/mL) in seminal plasma after intramuscular CAB-LA injections^*a*^^,*b*^

Visit/time point	*N*	Seminal plasma, median (IQR)	Blood plasma, median (IQR)	Seminal plasma ≤LLOQ, %	Median SP:BP ratio (IQR)	SP:BP ratio range
Day 3	21	12^*c*^ (6.5–23.5)	813 (451–2,541)	28.6%	0.01 (0.01–0.02)	0.01–0.04
Day 7	33	16^*c*^ (10–36)	1,457 (717–2,605)	27.3%	0.01 (0.01–0.02)	0.00–0.06
Day 28 (pre–2nd dose)	39	15.5^*c*^ (10–41.25)	1,367 (637–2,387)	7.7%	0.01 (0.01–0.02)	0.00–0.12
Day 84 (pre–3rd dose)	42	35 (23.25–49.75)	1,463 (1,012–2,347)	0%	0.02 (0.02–0.03)	0.01–0.07
Day 140 (pre–4th dose)	21	31 (18–48)	1,474 (1,155–2,228)	0%	0.02 (0.015–0.03)	0.01–0.05

^*a*^ Cabotegravir concentrations were quantified by liquid chromatography–tandem mass spectrometry (LC–MS/MS). The assay lower limit of quantification (LLOQ) was 4 ng/mL for seminal plasma; concentrations ≤LLOQ were considered quantifiable with >20% error and were therefore excluded from analysis. Quality control samples met standard bioanalytical acceptance criteria (5).

^*b*^ N, Number of participants with available concentrations.

^*c*^ Calculated on quantifiable samples: Day 3 (*N* = 15), Day 7 (*N* = 24), and Day 28 (*N* = 36).

In exploratory analyses, BMI was not significantly associated with the probability of detectable seminal CAB concentrations at days 3, 7, or 28 (all *P* > 0.1). At days 84 and 140, when all samples were quantifiable, BMI was not significantly associated with log-transformed seminal CAB concentrations in linear regression analyses (all *P* > 0.05). No HIV seroconversions were observed during the study period.

Consistent with prior evaluations of CAB distribution in genital tissues in PWH, our findings show that CAB exposure in seminal plasma is low but consistently proportional to plasma concentrations across repeated dosing intervals (1). This pattern mirrors the low tissue-to-plasma ratios observed in cervical, vaginal, and rectal compartments, in which CAB concentrations remain predictably linked to systemic levels despite limited absolute penetration (1). These observations reinforce the broader pharmacokinetic principle, demonstrated in healthy individuals, that CAB exhibits modest penetration into mucosal tissues but maintains a stable relationship between plasma and local concentrations.

Importantly, low total concentrations in seminal plasma should be interpreted in the context of markedly reduced protein binding in this matrix. However, in the present study, only total CAB concentrations were measured, and unbound concentrations were not directly quantified. A previous study in PWH receiving long-acting CAB plus rilpivirine showed that protein binding in seminal plasma is markedly lower than in blood (>99% in blood vs approximately 56% in semen), resulting in a considerably higher proportion of pharmacologically active unbound CAB (2). Based on the median seminal concentrations observed at steady state (31–35 ng/mL) and a previously reported estimated seminal protein binding of ~56% (2), the corresponding unbound concentrations would be approximately 13–15 ng/mL. These estimated values exceed reported EC_50_ values for CAB (~0.1–0.2 ng/mL) by more than two orders of magnitude and are also above commonly used pharmacodynamic targets such as 4× protein-adjusted IC_90_. Despite total seminal plasma concentrations representing a small fraction of blood levels, these indirect estimates suggest that unbound CAB concentrations in semen may exceed the EC_50_ by a wide margin. Together, these considerations suggest that local antiviral activity may be more closely related to unbound drug concentrations and local tissue microenvironment than to total concentrations alone (3). Protection conferred by CAB-LA PrEP is unlikely to depend solely on total seminal plasma concentrations. The long-acting formulation provides sustained systemic exposure with stable trough levels, supporting continuous drug distribution to genital tissues. Intracellular CAB exposure within semen-associated mononuclear cells and the class-specific pharmacokinetic/pharmacodynamic characteristics of InSTI may contribute to antiviral protection. As observed with other PrEP agents such as tenofovir, genital-fluid drug concentrations vary substantially across compounds and matrices and should therefore be interpreted cautiously when discussing mechanisms of protection. Sample availability varied across visits due to the real-world design and staggered enrollment, as not all participants returned for seminal sampling, and some had not yet completed follow-up at the time of analysis; however, BMI was not associated with seminal CAB exposure.

This study provides the first evidence that CAB reaches detectable concentrations in seminal plasma during CAB-LA PrEP. Despite low total levels, the observed proportionality between plasma and seminal concentrations, together with previously reported reduced protein binding in seminal fluid, supports the hypothesis that pharmacologically relevant unbound CAB exposure may occur in the male genital tract. These findings improve our understanding of CAB distribution across genital compartments and may help inform interpretation of compartment-specific pharmacology in HIV prevention.

## Data Availability

The data underlying this article will be shared on reasonable request from the corresponding author.
